# Factors associated with the utilization of community-based health services among older adults in China—an empirical study based on Anderson’s health behavior model

**DOI:** 10.1186/s12875-022-01697-9

**Published:** 2022-05-02

**Authors:** Wenyi Lin, Wanxia Yin, Dinghuan Yuan

**Affiliations:** 1 School of Public Administration, Jinan University, No.601, Huangpu Dadao Xi, Guangzhou, Guangdong Province China; 2 Common Prosperity and National Governance Institute, Jinan University, No.601, Huangpu Dadao Xi, Guangzhou, Guangdong Province China

**Keywords:** Community-based health services, Influencing factors, Anderson model, Healthy aging

## Abstract

Taking the modified Anderson health behavior model as the analysis framework and relying on 1136 empirical research data of *S* District in *Foshan* City, Guangdong Province of China, this study explores the influence of predisposing factors, enabling factors and need factors on the utilization of community-based health services among older adults in China. The results show that three variables have a significant impact on the use of family health services, which are whether the pension is the main source of living, income surplus, and major expenditure items. Seven variables have a significant impact on the use of preventive health services, which are household registration type, the basic endowment insurance coverage, the nature of the working unit before retirement, the self-rated health status, chronic diseases, self-care ability in daily life, and preventive health care needs.

## What is known about the topic

- Anderson health behavior model is widely used to predict and explain individual choice and

use of health services.

## What does this paper adds

- Some predisposing, enabling and need factors have a significant impact on the use of community-based health services among older adults in China.

## Introduction

China is the country with the largest elderly population in the world. By the end of 2019, there were 253.88 million people aged 60 and above, accounting for 18.1% of the total population [1]. With the rapid growth of the elderly population, elderly health issues require urgent attention. The *National Medical and Health Service System Planning Outline* (2015–2020) officially made it clear that the elderly care service should be fully integrated with the concept of health, and the support of health services should be strengthened. Besides, community-based health services should be developed, and the capacity of community-based health service organizations should be improved to provide daily care, chronic disease management, rehabilitation, health education and consultation, and traditional Chinese medicine health care services for older adults. Since 2016, contracts with general practitioner services have developed in urban areas of China [2]. Hospital institutions were encouraged to extend nursing services to households. The *Decision of the Fourth Plenary Session of the 19th Central Committee of the Communist Party of China* also pointed out that it is necessary to speed up the construction of an elderly care service system, in which the older adults are provided with home care, taken care of by community and supported by social services. A healthy aging strategy that focuses on improving the quality of life of the elderly, shortening the survival period with diseases, and extending healthy life expectancy is China’s way to actively and effectively respond to the rapid development of population aging.

China has established primary health care agencies in urban and rural communities. In urban China, community health care centers or stations are the main health care providers. In rural areas, township hospitals or village clinics are major health care providers. There were 970,036 primary health care agencies in urban and rural communities of China by the end of 2020. Among these primary health care agencies, there were 35,365 community health service centers or stations, 35,762 township hospitals, 608,828 village clinics, and 289,542 outpatient departments [1]. The primary health care agencies provide basic public health services for community residents. Basic public health services include disease and emergency prevention services, family health services, family rehabilitation, hospice and other medical services, health care services, and health education services. Chinese residents can get access to these public health services with support from government funding, universal health insurance coverage, and the basic public health service program [3]. In urban communities, community-based health services for older adults mainly include daily care, chronic disease management, rehabilitation, health education and consultation, and traditional Chinese medicine health care services. Some services such as health education and consultation services are free for older adults, and some services such as daily care are not free but the partial fees could be covered by health insurance. Some disadvantaged older adults can get access to these public services covered by social assistance programs or by community charitable endowment funds. In rural China, some developed areas have established community service provision systems providing similar types of community services as in urban areas. The less-developed rural areas mainly provide disease treatment services due to the paucity of public finance.

But at present, there are still few empirical studies on the efficiency and the effectiveness of community-based health service utilization. Taking the S District of *Foshan* City, Guangdong Province of China as an example, this study attempts to investigate the utilization of community-based health services and analyze factors affecting service utilization. Considering that specific community health service types are distinct in different provinces or cities in China, this study analyzes two basic types of community health service programs in terms of family health services and preventive health services in the S District of *Foshan* City.

### Literature review

The Anderson’s health behavior model was developed in the West countries, and had been widely tested [4–19]. In recent years, Anderson’s health behavior model has gradually been used to explain elderly care and medical decision-making behavior [6, 16, 20–23]. Some scholars have pointed out that the utilization rate of community health services in China is not high [24–27]. Chen and Chen claimed that the utilization of medical support services was the highest in Q street, *Putuo* District, Shanghai, but the utilization rate was only about 30% among all community residents with demands4.

Liu et al. conducted logistic regression analysis on the demand of health care service among older adults living in the community based on Anderson’s health behavior model, finding that age, the number of children, convenience for medical treatment, social support, the prevalence of chronic diseases, cognitive level, as well as the degree of fragility and depression were factors influencing older adults’ demand for health and nursing care services [16]. Based on the 2014 Chinese Longitudinal Healthy Longevity Survey (CLHLS), Chen and Wang observed that loneliness, community-based life care services, and chronic diseases were the influencing factors of unmet care needs [25]. Peng et al. used Anderson’s health behavior model to analyze the related factors affecting the use of long-term care services for the disabled elderly in China [22]. The study showed that enabling factors and need factors had a significant impact on the choice of services, while the predisposing factors had not passed the significance test.

In recent years, with the implementation of the policy of “Combining Medical Care with Nursing Care into the Community”, the health services are incorporated into the community-based care system. But health services are not exactly the same as existing services such as daily care services. The existing literature used the Anderson’s health behavior model to discuss health service utilization in hospitals, but ignored the health service utilization in the community settings. To fill the identified knowledge gap, this study adopts the modified Anderson health behavior model as the analysis framework to analyze a survey data from the elderly population in S District of *Foshan* City, Guangdong Province of China, aiming to identify key factors that affect older adults using two types of community-based health services.

This study uses the modified Anderson’s health behavior model as the theoretical framework, and classifies various individual factors that may affect the older adults’ use of community-based health services as independent variables. In order to better apply Anderson’s health behavior model in China, it is necessary to supplement the indicators. The social insurance variables (the insured situation of basic endowment insurance and basic medical insurance), the variable of working unit (the nature of the working unit before retirement), the traditional variable of family culture (the number of people living with the family and the living conditions with their families) are supplemented. This study investigates the influence of Chinese cultural tradition, social insurance system, and other factors on the utilization of community-based health services among older adults in China, which also makes the Anderson’s health behavior model more comprehensive.

### Research design

#### Data sources

The data used in this paper came from the community elderly care service survey conducted by S District Social Innovation Center entrusted by the Party Committee and Government Office of S District in *Foshan* City, Guangdong Province in 2017. The survey samples cover 4 street communities and 5 township communities in S District, and the respondents were elderly people over 60 years old. The sample was selected by using the multistage sampling method. The survey was conducted in person. A total of 1136 questionnaires were collected, of which 1061 were valid. The response rate was around 93.4%.

### Variable selection

#### Dependent variable

The dependent variables of this study are the use of community-based health services among older adults, including the use of family health services (family doctors, family beds, family appointment visits, family rehabilitation guidance and other personalized services) and the use of preventive health services (e.g., physical examination, chronic disease prevention, health records). The three dependent variables are binary variables. The elderly who used these community-based health services in the past 12 months were defined as 1, and those who did not use were defined as 0.

#### Independent variable

1. Predisposing factor.① Gender is a dummy variable (0 = female, 1 = male).② Age is a continuous variable. Considering the quadratic effect of age, the square of age was used as the independent variable.③ The household registration type was a multi-classification variable (1 = agricultural household registration, 2 = non-agricultural household registration, 3 = unified household registration), with “agricultural household registration” as the reference group.④ The education level was divided into three levels (1 = no schooling, 2 = primary school, 3 = junior high school and above), with “no schooling” as the reference group.⑤ Marital status was a dummy variable (0 = unmarried, 1 = married). This study classified unmarried, divorced and widowed into “unmarried” category.
2. Enabling factors.① Basic endowment insurance, as a classification variable, “retirement pension system of government and institution” was taken as a reference group.② Basic medical insurance, as a classification variable, with “public medical system of institutions” as the reference group.③ The nature of the working unit before employment or retirement was a dummy variable (0 = no working unit, including farming; 1 = having a working unit).④ The average monthly income was divided into six levels (1 = 1000 RMB and below, 2 = 1001–2000 RMB, 3 = 2001–3000 RMB, 4 = 3001–4000 RMB, 5 = 4001–5000 RMB, 6 = 5001 RMB and above), with “1000 RMB and below” as the reference group.⑤ Income surplus is divided into three levels (1 = make ends meet, 2 = basically enough, 3 = surplus), with “make ends meet” as the reference group.⑥ Whether pension is the primary source of living is a dummy variable (0 = no, 1 = yes).⑦ The main expenditure items were classified variables, with “daily diet and clothing consumption” as the reference group.⑧ The number of family members living together was a continuous variable.⑨ Living conditions were classified variables (1 = living with husband, wife and other family members, 2 = living with family members without the respondent’s husband and wife, 3 = living with husband and wife only, 4 = living alone).
3. Need factors.① Health status: the data was obtained through the self-rated health status of the interviewees. There were five options in the questionnaire: “very healthy, relatively healthy, general, relatively unhealthy and very unhealthy”. In this study, “very healthy and relatively healthy” was defined as “good health”, which was assigned as 1, and the remaining three options were defined as “poor health” with a value of 0.② Patients with chronic disease were dummy variables (0 = no, 1 = yes).③ Self-care ability of daily life was a dummy variable (0 = no difficulty, 1 = difficulty). Eight questions were set up in the questionnaire to ask the respondents whether they could independently carry out daily activities (ADL: including eating, bathing, defecation and indoor activities) and instrumental daily activities (IADL: including cooking, washing clothes, shopping, taking public transportation) to assess the living ability of the respondents. If any of the activities of daily life were difficult (choose “need some help” or “completely”), it was defined as “having difficulty in self-care ability in daily life”.④ The need for health care for the elderly was a dummy variable (0 = no need, 1 = need).

### Statistical analysis

The dependent variable in this paper is a binary variable, which is suitable for the binary logistic regression model. Thus, three binary logistic regression models were used to predict the utilizations of family health services and preventive health services. This study used descriptive statistics to described the characteristics of all variables. Thereafter, two Chi-Square tests were used to examine the relationship between service demand and service utilization. And then two regression models were employed to predict service utilization. The statistical software package SPSS 24.0 (International Business Machines Corp: Beijing, China) was applied for all data analyses.

## Results

### Descriptive statistics

The basic information of the sample is shown in Table 1.The situation of elderly people using family health services in terms of family doctors, family beds, family appointment visits, rehabilitation guidance, and other family medical and health services was not well. The demand rate was 20.7% among all respondents, while the utilization rate was only 0.8% in total sample. The demand rate of preventive health care services such as physical examination, chronic disease treatment, and health records reached 66.6%, and the utilization rate was 52% in total sample.

**Table 1:** Descriptive statistics of variables (*n* = 1061)

	Variable	Mean (SD) or Per cent
Dependent variable	Family health medical services (Use)	0.8%
	Preventive health services (Use)	52%
Predisposing factors	Gender (Male)	44.4%
	Age (Mean / SD)	71.48/1.705
	Household registration	
	Agricultural household registration	63.7%
	Non agricultural household registration	20%
	Unified household registration	16.3%
	Education level	
	No formal education (illiterate)	18.9%
	Primary school	58.8%
	Junior high school and above	22.3%
	Marital status (married)	72.7%
Demand factors	Self rated health (good health)	50.2%
	Suffering from chronic diseases (yes)	57.4%
	Self care ability in daily life (yes)	90.2%
	Demand for health-related services	
	Family health service (No)	79.3%
	Preventive health care services (No)	33.4%
Enabling factors	The nature of the working units before the current employment or retirement (Having working units)	50.40%
	Basic endowment insurance	
	Retirement pension system of government and institution	4.5%
	Basic endowment insurance for urban employees	34.5%
	Basic endowment insurance for urban and rural residents	40.9%
	Did not participate in any social endowment insurance	20.1%
	Basic medical insurance	
	Public medical system of government and institution	3.9%
	Basic medical insurance for urban employees	32.2%
	Basic medical insurance for urban and rural residents	55.9%
	No social medical insurance	8%
	Average monthly income	
	1000 RMB and below	31.3%
	1001–2000 RMB	28.7%
	2001-3000RMB	20.5%
	3001-4000RMB	12.9%
	4001-5000RMB	3.3%
	5001RMB and above	3.2%
	Income surplus	
	Make ends meet	24.9%
	Basically enough	64.7%
	There is a surplus	10.5%
	Pension as the main source of living (Yes)	51.5%
	Major expenditure items	
	Daily diet and clothing consumption	75.6%
	Water, electricity, property, transportation and communication	5.3%
	See a doctor / buy health products	16.5%
	Rehabilitation nursing / professional nursing service fee	0.7%
	Domestic service	0.4%
	Cultural and entertainment consumption	1.5%
	Number of CO residents (Mean / SD)	4.15/0.06
	Living conditions	
	Living with husband, wife and other family members	50.5%
	Living with family members without the respondent’s husband and wife	26.8%
	Living with husband and wife only	15.2%
	Living alone	7.5%

### Chi-Square tests

Table 2 indicates the relationship between service demand and service utilization. Among the respondents with the demand for family health service, only 4% of them had used this type of service. 75.3% of respondents with demand for preventive health care services had used this type of service. The relationship between family health service demand and the utilization is significant (χ 2 = 34.462, *P* < 0.001). The preventive health care service demand is also significantly associated with service utilization (χ 2 = 492.916**,** *P* < 0.001).

**Table 2:** Relationship between health service demand and service utilization (*n* = 1061)

	Family health medical services		Preventive health services	
	Not having demand	Having demand	Not having demand	Having demand
Did not use	885	224	355	186
Use	0	9	19	566
Chi-square	34.462***		492.916***	

*p < 0.1, **p < 0.05, ***p < 0.01

### Regression analysis

Table 3 reports the diagnosis results of multicollinearity of independent variables. The variance expansion factor (VIF) of each variable is far less than 10, indicating that the problem of multicollinearity between independent variables is not serious.

**Table 3:** Collinearity diagnosis

Independent variable	Family health service	Preventive health services
	VIF	VIF
Gender	1.198	1.199
Age	1.027	1.027
Registered residence	1.350	1.350
Marital status	1.387	1.384
Education level	1.367	1.366
Basic endowment insurance	3.493	3.479
Basic medical insurance	2.779	2.775
Nature of working unit before retirement	1.673	1.657
Average monthly income	2.019	2.000
Pension as the main source of living	1.720	1.721
Income surplus	1.353	1.351
Major expenditure items	1.083	1.088
Number of co-residents	2.039	2.027
Living conditions	2.299	2.292
Self rated health	1.344	1.353
Suffering from chronic diseases	1.230	1.284
Self care ability in daily life	1.142	1.132
Demand for family health services	1.053	
Demand for preventive health services		1.091

Table 4 presents the results of regression models. The three logistic regression models constructed in this study all passed the significance test, that is, Model 1 (χ 2 = 67.486, *P* < 0.001), Model 2 (χ 2 = 629.777, *P* < 0.001), and Model 3 (χ 2 = 487.097, *P* < 0.001) were statistically significant.

**Table 4:** Logistic regression result

Variable	Family health service		Prevention health service	
	β value	Exp(B)	β value	Exp(B)
Constant	−59.995	0.000	−1.577	0.207
**Gender** (Reference group: Female)	2.266	9.643	−0.122	0.885
**Age**	0.902	2.464	0.009	1.009
**Age squared**	−0.006	0.994	0	1
**Household registration** (Reference group: Agricultural household registration)				
Non agricultural household registration	−2.45	0.086	−0.702***	0.496
Unified household registration	− 22.152	0.000	−0.791***	0.453
**Marital status** (Control group: Unmarried)	0.042	1.043	0.228	1.257
**Education level** (Reference group: No formal education)				
Primary school	1.915	6.788	0.054	1.055
Junior high school and above	−15.743	0.000	−0.304	0.738
**Basic endowment insurance** (Reference group: Retirement pension system of government and institution)				
Basic endowment insurance for urban employees	−11.606	0.000	−1.941	0.144
Basic endowment insurance for urban and rural residents	−8.958	0.000	−2.461*	0.085
Did not participate in any social endowment insurance	−4.544	0.011	−2.511**	0.081
**basic medical insurance** (Reference group: Public medical system of government and institution)				
Basic medical insurance for urban employees	3.231	25.313	0.354	1.424
Basic medical insurance for urban and rural residents	2.544	12.73	0.157	1.17
No social medical insurance	5.385	218.195	0.67	1.954
**Nature of unit before retirement** (Reference group: No working unit)	−0.212	0.809	0.473*	1.605
**Average monthly income**	1.962	7.11	−0.134	0.875
**Pension as the main source of living** (Reference group: No)	3.556*	35.04	−0.263	0.769
**Income surplus** (Reference group: Make ends meet)				
Basically enough	−4.45**	0.012	−0.167	0.846
There is a surplus	−13.527	0	−0.085	0.918
**Major expenditure items** (Reference group: Daily diet and clothing consumption)				
Water, electricity, property, transportation and communication	6.647**	770.587	−0.265	0.767
See a doctor / buy health products	3.982**	53.63	0.393	1.482
Rehabilitation nursing / professional nursing service fee	−21.364	0	−20.758	0
Domestic service	−14.113	0	−0.748	0.473
Cultural and entertainment consumption	8.52	5016.197	0.602	1.826
**Number of CO residents**	−0.365	0.694	0.053	1.054
**Living conditions** (Reference group: Living with husband, wife and other family members)				
Living with family members without the respondent’s husband and wife	0.576	1.779	0.172	1.187
Living with husband and wife only	−4.098	0.017	0.247	1.281
Living alone	3.062	21.376	−0.224	0.799
**Self rated health**(Reference group: Poor health)	−0.75	0.721	−0.355*	0.701
**Suffering from chronic diseases** (Reference group: No)	0.848	2.334	0.397**	1.488
**Self care ability in daily life** (Reference group: No difficulty)	2.451	11.595	−1.052***	0.349
**Family health service demands** (Reference group: no demand)	21.391	1.95E+ 09	/	/
**Preventive health service demands** (Reference group: no demand)	/	/	4.357***	78.03
**Chi-square (df)**	67.486 (32)***		629.777 (32)***	

*p < 0.1, **p < 0.05, ***p < 0.01

#### The influence of predisposing factors

The two models showed that the type of household registration had a significant influence on the use of preventive health care services. The results showed that compared with agricultural household registration, the elderly with non-agricultural household registration (β = − 0.702, *P* < 0.01) and unified household (β = − 0.791, *P* < 0.01) were less likely to use preventive health care services. There was no significant effect on the use of family health services by the elderly.

#### The influence of enabling factors

The empirical results showed that the basic endowment insurance coverage of the elderly, the nature of the working unit before retirement, average monthly income, whether the pension is the main source of living, income surplus, major expenditure items, and living conditions had a significant impact on whether the elderly use community-based health services. In terms of the use of family health services, compared with the elderly who did not rely on pension as the main source of living, the elderly people with pension as the main source of income were more likely to use family health services (β = 3.556, *P* < 0.1). The effect of income surplus on the use of family health services is negative, that is, the elderly with “ make ends meet “ are more likely to use family medical services than those with “basically sufficient income” (β = − 4.55, *P* < 0.05). The possible explanation for this statistical result is that there is a reverse causal relationship between the income surplus and whether the elderly use family health services. Because of the large proportion of medical expenditures and the high amount of medical expenditures, this part of the elderly “cannot make ends meet”. Taking the elderly whose main expenditure items were daily diet and clothing consumption as the reference group, the elderly whose main expenditure items were “water, electricity, property, transportation and communication” (β = 6.647, *P* < 0.05) and “seeing a doctor / purchasing health care products” (β = 3.982, *P* < 0.05) were more likely to use family health services.

Concerning the use of preventive health care services, compared with the elderly who enjoyed the retirement pension treatment of government institutions, the elderly who participated in the basic endowment insurance of urban and rural residents (β = − 2.461, *P* < 0.1) and those who did not participate in any social endowment insurance (β = − 2.511, *P* < 0.05) were less likely to use preventive health care services. At present, the pension of government institutions is higher than that of employees, and the pension of employees is higher than that of residents. The level of pension to a large extent represents the economic ability of the elderly, thus affecting whether the elderly groups use preventive health care services. Compared with the elderly who enjoy the retirement treatment of government institutions, the elderly who did not participate in any social endowment insurance were the least likely to use preventive health care services. Before retirement, the elderly with working units were more likely to use preventive health services than those without (including farming) (β = 0.473, *P* < 0.1). Similar to the above explanation of the possibility of participating in the basic endowment insurance, the old people who have a working unit before retirement enjoy the retirement treatment of government and public institutions or receive employee pension, which may be more secure in terms of source of living than the elderly without a unit (including farming). The difference like the working units before retirement is also reflected in the aspect of health security. The retirees of some working units enjoy the benefits of public medical treatment of government institutions or basic medical insurance for urban workers, while the retirees without a unit can only participate in the basic medical insurance for urban and rural residents, or even not participate in any basic medical insurance. Regardless of whether it is outpatient or inpatient, the level of protection and strength of the above two is quite different. Therefore, the nature of the pre-retirement working unit is more likely to affect the elderly’s attitude toward seeking medical care in the economic dimension, as well as their medical actions-whether to use preventive health services.

#### The influence of need factors

The empirical results showed that the health status of need factors, chronic diseases, self-care ability of daily life, and service need had a significant influence on whether the elderly use community-based health services. In terms of preventive health care services, the impact of self-rated health status and self-care ability of daily life on the use of services is negative, that is, the elderly with poor health status are more likely to use preventive health care services than those with good health status (β = − 0.355, *P* < 0.1), which is similar to the research conclusion of Chen and Ma [14]. The worse the physical condition of the elderly, the higher the demand for health services, thus, the more likely they are to use preventive health services. The elderly with no difficulties in daily life were more likely to use preventive health services than those with difficulties (β = − 1.052, *P* < 0.01). The possible explanation for this statistical result is that the elderly people who take care of themselves are more sensitive and free in walking, daily activities, eating, and defecation, and so on, so they take the initiative to participate in the physical examination and chronic diseases provided by the community. Another possibility is that people using preventive health services are healthier and less likely to have difficulties in daily life.

Compared with the elderly without chronic diseases, the elderly with chronic diseases used more preventive health services (β = 0.397, *P* < 0.05). With the decline of health status, the elderly with chronic diseases have a stronger demand for prevention and health care, which is consistent with the research of Li [11]. The disease is a major adverse factor that perplexes the elderly in their later years, and the elderly diseases are mainly cardiovascular and cerebrovascular diseases (hypertension, coronary heart disease, and cerebral apoplexy), diabetes, and other common chronic diseases. The elderly with chronic diseases are more likely to use community preventive health care services because they need to pay attention to and investigate their own health problems at all times, so as to reduce the potential risks. The elderly with preventive care needs were more likely to use services than those without (β = 4.357, *P* < 0.01).

In terms of family health services, although the enabling factors had not passed the significance test, it is found that the elderly with poor health status, chronic diseases, difficulties in daily life and family health service demand were more likely to use family health services. With the growth of age, the physical health status of the elderly gradually deteriorates, and the risk of chronic diseases continues to increase. In addition, daily self-care ability is declining and health problems are prominent. As a result, the possibility of using family health services will increase.

## Discussion

The results show that among the 18 independent variables, three variables have a significant impact on the use of family health services, which are whether the pension is the main source of living, income surplus and major expenditure items. Seven variables have a significant impact on the use of preventive health services, which are household registration type, the basic endowment insurance coverage, the nature of the working unit before retirement, the self-rated health status, chronic diseases, self-care ability in daily life, and preventive health care needs., Several predictors such as social support, the prevalence of chronic diseases, and degree of fragility are similar to the findings of Liu et al. [16] who used Andersen’s model to predict community health and nursing services’ demands. Based on the findings, this study provides the following suggestions for improving the use efficiency of community-based health services.

First, the government should pay attention to the economic income of the elderly, and improve the health care service subsidy system. The results show that economic variables such as basic endowment insurance, average monthly income, whether the pension is the main source of living, income surplus, main expenditure items, and other economic variables have a significant impact on whether the elderly use health care services. Some lower-income elderly people may be difficult to purchase and use community-based health services because of their insufficient ability to pay. The government should further improve the classification of the elderly’s health status and economic income evaluation mechanism, formulate detailed and reasonable service subsidy standards, and provide appropriate subsidies for the purchase of health and elderly services for low-income elderly people who do have a demand for health and elderly care services and lack financial capacity. By doing this, service provision organizations can transform potential demands into actual demands and increase service utilization rate [11]. At the same time, differentiated government subsidies and the scope of government-purchased services can be established according to different types and levels of health and elderly care services, and the threshold for enjoying government-purchased services can be appropriately relaxed, so that more needy elderly people can enjoy free services.

Second, the government should establish a unified evaluation mechanism of community home-based care services. The results of this study show that the utilization rate of community-based health services in S District is not high, which may be due to the insufficient supply of health care services and the mismatch between supply and need. Logistic regression results confirmed that there were differences in the influencing factors of different types of service use, and the elderly with health care service needs were more likely to use health service items. Therefore, the service supply should respond to the health demands of the elderly to achieve the balance of supply and need of community-based health services. It is necessary to establish a unified health status classification and needs assessment mechanism, and establish an elderly information database to comprehensively integrate and evaluate the information of the elderly, such as age, physical health status, self-care ability, family situation, and economic status. According to the health status and needs assessment results of the elderly, accurately identify service needs, flexibly and accurately provide different kinds of community-based health services for different older adults, and allocate community medical and health resources and pension service resources fairly and effectively. Service providers should accurately identify service needs based on the health status of the elderly and the results of needs assessments, flexibly and accurately delivering differentiated and personalized community health and elderly care services to service targets. Community health resources and elderly care service resources should be allocated fairly and effectively [9]. At the same time, we will continue to expand elderly care services based on the health needs of the elderly, deepen the quality of services, and further enhance the sense of acquisition and satisfaction of the elderly.

Last but not the least, the Chinese government should strengthen the capacity of community health service provision agencies in urban and rural areas. The Chinese government should further promote the development mode of combining medical care with nursing care into the community, actively develop healthy elderly care service projects, and improve the professionalism of health care services and the accessibility and convenience of elderly access to services. At the community level, we should strengthen the publicity of health care services through multiple channels, improve the overall cognitive level of the elderly on the health care service projects, cultivate the positive aging concept of the elderly, and enhance the cognition, acceptance and participation of the elderly in the community service model.

Although the survey sample is representative of the elderly population in the study area, the findings cannot be generalized to all aged population in China considering community health services are heterogeneous in different cities. Regarding the representativeness of Guangdong province, the economic and social development of S District of *Foshan* City is lined up in front of Guangdong Province, thus S District can represent the developed areas of Guangdong Province, but cannot represent the whole Guangdong Province. In addition, this study did not include all types of community health services for older adults due to the lack of data. Nevertheless, this study contributes to understanding the application of Andersen’s model in community-based health service utilization among older adults in the Chinese context.

## Data Availability

The datasets used and/or analysed during the current study available from the corresponding author on reasonable request.

## References

[1] China Statistical Bureau (2021). China Statistical Yearbook.

[2] Liu Z, Tan Y, Liang H, Gu Y, Wang X, Hao Y, Hao C (2019). Factors Influencing Residents’ Willingness to Contract With General Practitioners in Guangzhou, China, During the GP Policy Trial Phase: A Cross-Sectional Study Based on Andersen’s Behavioral Model of Health Services Use. Inquiry.

[3] Li X, Lu J, Hu S, Cheng K, De Maeseneer J, Meng Q (2017). The primary health-care system in China. Lancet.

[4] Adane M, Mengistie B, Mulat W, Kloos H, Medhin G (2017). Utilization of health facilities and predictors of health-seeking behavior for under-five children with acute diarrhea in slums of Addis Ababa, Ethiopia: a community-based cross-sectional study. J Health Population and Nutrition.

[5] Broyles RW, Narine L, Brandt EN, Biard-Holmes D (2000). Health risks, ability to pay, and the use of primary care: is the distribution of service effective and equitable?. Prev Med.

[6] Fields N, Richardson V, Schuman D (2017). Marital Status and Persons With Dementia in Assisted Living: An Exploration of Length of Stay. Am J Alzheimer s Dis Other Dementias.

[7] Freidoony L, Ranabhat CL, Kim CB, Kim CS, Ahn DW, Doh YA (2018). Predisposing, enabling, and need factors associated with utilization of institutional delivery services: A community-based cross-sectional study in far-western Nepal. Women Health.

[8] Green SE (2004). The impact of stigma on maternal attitudes toward placement of children with disabilities in residential care facilities. Soc Sci Med.

[9] Gu L, Rosenberg MW, Zeng J (2017). Changing caregiving relationships for older home-based Chinese people in a transitional stage: Trends, factors and policy implications. Arch Gerontol Geriatr.

[10] Houle L, Salmoni A, Pong R, Laflamme S, Viverais-Dresler G (2001). Predictors of Family Physician Use Among Older Residents of Ontario and An Analysis of the Andersen-Newman Behavior Model. Can J Aging La Revue Canadienne du vieillissement.

[11] Hsieh YL, Lee FH, Chen SC, Tang JS (2019). Factors associated with the intention to use adult preventive health services in Taiwan. Public Health Nurs.

[12] Iecovich E, Carmel S. Differences Between Users and Nonusers of Day Care Centers Among Frail Older Persons in Israel. J Appl Gerontol. 2010;29. 10.1177/0733464810372771.

[13] Nivestam A, Westergren A, Petersson P, Haak M (2020). Factors associated with good health among older persons who received a preventive home visit: a cross-sectional study. BMC Public Health.

[14] Petrovic K, Blank TO (2015). The Andersen–Newman Behavioral Model of Health Service Use as a conceptual basis for understanding patient behavior within the patient–physician dyad: The influence of trust on adherence to statins in older people living with HIV and cardiovascular disease. Cogent Psychol.

[15] Pianori D, Maietti E, Lenzi J, Quargnolo M, Guicciardi S, Adja KYC, Toth F (2020). Sociodemographic and health service organizational factors associated with the choice of the private versus public sector for specialty visits: Evidence from a national survey in Italy. PLoS One.

[16] Sunil TS, Rajaram S, Zottarelli LK (2006). Do individual and program factors matter in the utilization of maternal care services in rural India?: a theoretical approach. Soc Sci Med.

[17] Tolera H, Gebre-Egziabher T, Kloos H (2020). Using Andersen's behavioral model of health care utilization in a decentralized program to examine the use of antenatal care in rural western Ethiopia. PLoS One.

[18] Travers JL, Hirschman KB, Naylor MD (2020). Adapting Andersen’s expanded behavioral model of health services use to include older adults receiving long-term services and supports. BMC Geriatr.

[19] Yoo JP, Kim H, Han KH (2020). Community-based social service utilization of marriage migrants in Korea: Focusing on differences by women's country of origin. Soc Sci J.

[20] Peng XZ, Song JJ, Huang JK (2017). Determinants of Long-Term Care Services among Disabled Older Adults in China: A Quantitative Study based on Andersen's Behavioral Model. Popul Res.

[21] Li CY, Zhang HP (2018). Analysis of the elderly's choice of the mode of home—based communal medical care combined with aging services in ethnic minority areas and its influencing factors in the perspective of Anderson's behavior model. J Yunnan Minzu Univ (Social Sciences).

[22] Sun LY, Su CH, Du QY (2019). Factors on Rural Elders’ Decision-Making Behavior of Elderly Caring. Popul Dev.

[23] Chen N, Wang CQ (2020). Determinants of unmet need among disabled elderly living alone in China. Modern Prev Med.

[24] Li F, Wang YY (2016). Utilization status and Influencing Factors of Community Home-based care Services——Based on a survey in Gulou District, Nanjing city. Popul Soc.

[25] Chen YY, Chen HL (2017). The Disparity between demand and use: Reflections on delivery of Community Home-based Care Services. Zhejiang J.

[26] Lu JW, Li F (2018). Empirical Research on the Current Situation of the Utilization and Satisfaction of the Market for Home-based Care Services—Based on the Survey of Four Cities in Jiangsu Province. Soc Welfare (Theoretical edition).

[27] Hu YH, Li GR, Zhong XY (2019). Using Status and Influencing Factors of Home:Based Care Services for The Elderly in Putian City. Northwest Popul.

